# Hypoxia Inducible Factor Pathway and Physiological Adaptation: A Cell Survival Pathway?

**DOI:** 10.1155/2015/584758

**Published:** 2015-09-27

**Authors:** Hemant Kumar, Dong-Kug Choi

**Affiliations:** Department of Biotechnology, Konkuk University, Chungju 380-701, Republic of Korea

## Abstract

Oxygen homeostasis reflects the constant body requirement to generate energy. Hypoxia (0.1–1% O_2_), physioxia or physoxia (∼1–13%), and normoxia (∼20%) are terms used to define oxygen concentration in the cellular environment. A decrease in oxygen (hypoxia) or excess oxygen (hyperoxia) could be deleterious for cellular adaptation and survival. Hypoxia can occur under both physiological (e.g., exercise, embryonic development, underwater diving, or high altitude) and pathological conditions (e.g., inflammation, solid tumor formation, lung disease, or myocardial infarction). Hypoxia plays a key role in the pathophysiology of heart disease, cancers, stroke, and other causes of mortality. Hypoxia inducible factor(s) (HIFs) are key oxygen sensors that mediate the ability of the cell to cope with decreased oxygen tension. These transcription factors regulate cellular adaptation to hypoxia and protect cells by responding acutely and inducing production of endogenous metabolites and proteins to promptly regulate metabolic pathways. Here, we review the role of the HIF pathway as a metabolic adaptation pathway and how this pathway plays a role in cell survival. We emphasize the roles of the HIF pathway in physiological adaptation, cell death, pH regulation, and adaptation during exercise.

## 1. Introduction 

Hypoxia inducible factor (HIF) senses and coordinates cellular responses to hypoxia. HIF is a heterodimer consisting of one of three alpha (*α*) subunits and a beta (*β*) subunit. HIF-*β* is constitutively expressed, whereas HIF-*α* is induced by hypoxia. HIF-1*α* is the most well-established member of the HIF family; the other two members of the basic–helix–loop–helix-PAS (bHLH-PAS) superfamily are HIF-2*α* (endothelial PAS domain protein 1 or HIF1*α*-like-factor), which also stabilizes hypoxia and binds to the aryl hydrocarbon receptor nuclear translocator (ARNT) [1, 2] and HIF-3*α* [3]. HIF-1*α* is a transcriptional activator and a master regulator for the expression of genes involved in the response to hypoxia in most mammalian cells [4]. HIF-1*α* is prolyl hydroxylated at P402 and P564 in its oxygen-dependent degradation domain (ODD) under normoxic conditions. This leads to binding of the HIF-*α* unit to the E3 ubiquitin ligase von Hippel-Lindau protein (pVHL) at L574 for degradation [5, 6]. Homologous proline and leucine residues in HIF-2*α* also play similar roles [7]. HIF-1/2*α* transactivation activity is further inhibited by asparaginyl hydroxylation under normoxic conditions [8]. Low oxygen tension stabilizes the *α*-subunit and leads to nuclear translocation, formation of a dimer with HIF-1*β*, and recruitment of transcriptional coactivators [9]. This complex binds to an enhancer domain of the hypoxia responsive element (HRE) located either at the 5′ or 3′ region of target genes, including heme oxygenase-1, vascular endothelial growth factor (VEGF), glucose transporter (GLUT)-1, and GLUT-4 [10]. Interestingly, HIF-1*α* also upregulates glycolytic enzymes and glucose transporters, allowing cells to depend more heavily on glycolysis for energy [11], suggesting that HIF-1*α* also modulates aerobic metabolism. The vast majority of HIF target genes are regulated by HIF-1*α*, whereas exclusively HIF-2*α*-dependent genes are scarce and cell type-dependent [12–15]. Most reports show that HIF-1*α* and HIF-2*α* are master regulators of the transcriptional response to hypoxia, but the role of HIF-3*α* under hypoxic conditions is far less clear. HIF-3*α* is the newest member of the family and may be more restricted than the other HIF subunits [3]. HIF-3*α* mRNA may also be activated by hypoxia [16]. Evidence suggests that HIF-3*α* may be functionally distinct. HIF-3*α* sequence has relatively low identity with HIF-1*α*/2*α* [3]. HIF-1*α* and HIF-2*α* have two terminal transactivation domain (TADs) [1, 10], whereas HIF-3*α* has only one TAD. HIF-3*α* has a unique leucine zipper domain and an LXXLL motif [3], and these unique structural features are evolutionarily conserved [17]. HIF-3*α* has multiple splice variants, and the inhibitory PAS domain protein is the most studied, which is a truncated protein and a dominant-negative inhibitor of HIF-1*α* [18]. HIF-1*α* and HIF-2*α* have 48% amino acid sequence identity and similar protein structures, but are nonredundant and have distinct target genes and mechanisms of regulation. Interestingly, HIF-1*α* appears the most active isoform during short periods (2–24 h) of intense hypoxia or anoxia (<0.1% O_2_) in some cell lines, whereas HIF-2*α* is active under mild or physiological hypoxia (<5% O_2_), and continues to be active even after 48–72 h of hypoxia [19]. Thus, in some contexts, HIF-1*α* plays key role in initial response to hypoxia whereas HIF-2*α* drives the hypoxic response during chronic hypoxic exposure [19, 20]. This HIF “switch” results between HIF-1*α* and HIF-2*α* suggests physiological and pathological adaptation required to adapt for cell survival. Interestingly, these isoform plays opposite but balancing roles during the hypoxic response under both physiological and pathophysiological conditions in some cells.

## 2. Prolyl Hydroxylases: Oxygen Sensors

Prolyl hydroxylases (PHDs) [1, 2 and 3] are evolutionary conserved oxygen sensors in metazoans. These dioxygenases were discovered after confirming that oxygen-dependent enzymatic activity covalently modifies an HIF-1*α* domain known as the ODD domain [21–23]. PHDs require 2-oxoglutarate as a cosubstrate, molecular oxygen, and iron liganded by two histidine and one aspartic acid residues to function as hydroxylases. PHDs play a key role in the HIF-mediated hypoxia signaling pathway, which facilitates cell survival and adaptation in response to capricious environmental oxygen levels [24]. Selective knockdown of PHDs enhances HIF-dependent gene expression* in vitro *[25]. PHDs lose their activity under hypoxic conditions, leading to accumulation and nuclear translocation of HIF-*α* and activation of HIF target genes by binding to HREs [26]. The first identified function of PHDs was to hydroxylate human HIF-1*α* h-subunits at Pro402 and Pro564 under normoxic conditions, resulting in their recognition, pVHL ubiquitylation, and degradation by 26S proteasomes. The PHD catalytic domain recognizes a specific LXXLAP motif in the ODD of the HIF-*α* subunits [27]. PHD1–3 have near ubiquitous tissue expression; PHD-2 is generally the most abundant isoform, with the exception of the testis, where PHD-1 is the most highly expressed isoform, and the heart, where PHD-3 expression predominates [28]. Loss of PHD-1 lowers skeletal muscle and liver oxygen consumption by reprogramming glucose metabolism from primarily oxidative to more anaerobic ATP production, suggesting selective loss of PHD-1-induced hypoxia tolerance [29, 30]. Knockdown of the Phd-2 gene, but not those of Phd-1 and Phd-3, results in embryonic death due to placental and heart defects, suggesting that PHD-2 is essential during mouse embryogenesis [31]. Interestingly, broad spectrum conditional Phd-2 knockout in adult mice leads to hyperactive angiogenesis, angiectasia, and congestive heart failure, suggesting that PHD-2 is a major negative regulator of vascular growth [32, 33]. Furthermore, PHD-3 is required for proper anatomical and physiological integrity of the system, as loss of PHD-3 results in abnormal sympathoadrenal development and systemic hypotension [34]. Targeted deletion of the PHD-3 gene increases angiogenesis and preserves cardiac function by stabilizing HIF-1*α* after infarction, suggesting a potential target for pharmacological management of ischemic myocardial disease [35].

## 3. Hypoxia and Reactive Oxygen Species

Oxygen scarcity leads mitochondria to produce reactive oxygen species (ROS) thereby giving alert to cells to the shortage. ROS describe a range of molecules and free radicals (chemical species with one unpaired electron) derived from molecular oxygen. The mitochondrial electron transport chain contains several redox centers that may leak electrons to oxygen, constituting the primary source of superoxide (precursor of most ROS) in most tissues. Superoxide anion is produced both enzymatically and nonenzymatically* in vivo*. Enzymatic sources include NADPH oxidases [36, 37] as well as cytochrome P450-dependent oxygenases [38]. Proteolytic conversion of xanthine dehydrogenase to xanthine oxidase provides another enzymatic source of both superoxide and H_2_O_2_ (and therefore constitutes a source of OH^∙^) and has been proposed to mediate deleterious processes* in vivo* [39]. Nonenzymatic production of superoxide occurs when a single electron is directly transferred to oxygen by reduced coenzymes or prosthetic groups or by xenobiotics previously reduced by enzymes. According to the equation

(1) $$\begin{array}{c} \frac{d\left[superoxide\right]}{dt}=k\left[O_{2}\right]\left[R^{∙}\right], \end{array}$$

where R^∙^ is an electron donor, the rate of superoxide formation increases with oxygen concentration under normobaric and hyperbaric conditions [40]. As predicted by (1), mitochondrial production of superoxide anion should increase with oxygen concentration, but the proportion of superoxide anion is less than predicted in tissue exposed to atmospheric oxygen [41, 42]. ROS activate the HIF pathway during hypoxia leading to stabilization of the HIF(s) *α*-isoform. The generation of ROS should decrease with hypoxia according to (1), yet many reports show increased oxidative stress under moderately hypoxic conditions [43–45] but not under severe hypoxic conditions [44]. Moreover, substantial evidence indicates a role for the functional respiratory chain in the generation of ROS under hypoxic conditions. A mutation in the respiratory chain [45, 46] or complex 1 inhibitors [47] prevent stabilization of HIF under hypoxic conditions. More research is needed to clarify the paradoxical ROS formation in the response of tissues to hypoxia.

## 4. Hypoxia and Mitochondrial Respiration

Mitochondria are the largest consumers of cellular O_2_ and are likely candidates for the location of a cellular oxygen sensor. Mitochondria are both targets and important sources of free radicals [48]. Most vital intracellular processes, including blood vessel maintenance, heart contractibility, lung functioning, and neurotransmitter and hormonal support require mitochondria. Mitochondria are the source of ATP and maintain oxygen homeostasis at both the systemic and cellular levels [49]. Initial evidence supporting mitochondria as an oxygen sensor came with the discovery that mitochondrial-depleted Hep3B cells fail to respire and activate mRNAs of erythropoietin, glycolytic enzymes, or VEGF during hypoxia. These cells also fail to increase the generation of ROS during hypoxia, suggesting that mitochondrial (mt) ROS trigger hypoxia-induced transcription [45]. Mitochondria are implicated in multiple HIF-dependent and independent pathways through production of mtROS. Interestingly, decreasing mtROS levels with mitochondrial inhibitors or ROS scavengers prevents stabilization of HIF-1*α* under hypoxic conditions, suggesting that ROS are important for this effect [45–47]. Interestingly, murine embryonic cells lacking cytochrome* c*, and therefore mitochondrial activity, have impaired cellular oxygen sensing, which prevents stabilization of HIF-1*α* and HIF-2*α* under hypoxic conditions, suggesting that mtROS act upstream of PHD to regulate HIF-1*α* and HIF-2*α* [50]. Mitochondrial complex III is required for hypoxia-induced ROS production and cellular oxygen sensing [51, 52]. Similarly, embryonic stem cells lacking cytochrome *c* fail to stabilize HIF-1*α* during hypoxia, as loss of cytochrome *c* leads to complete reduction of cytochrome c1 and the rieske iron-sulfur protein, which inhibit transfer of electrons from ubiquinol at the Q_o_ site [50]. The complex III Q_o_ site is necessary to increase cytosolic ROS under hypoxic conditions, which inhibits PHDs from degrading the HIF-1*α* protein [53]. Additionally, exogenous H_2_O_2_ or mutations leading to H_2_O_2_ accumulation stabilize HIF-1*α* during normoxia [52, 54]. In accordance, antioxidants abolish the hypoxic HIF response, suggesting that generation of mtROS is responsible for propagating the hypoxic signal [45].

## 5. Physiological Adaptation by HIF(s)

### 5.1. Physiological Importance of HIF(s)

The role of the HIF pathway has been demonstrated under hypoxic conditions but it also plays an important role in normoxia. The physiological roles of HIF-1*α* and HIF-2*α* are not known in detail. Hif1*α* ^−^/^−^ knockout mice have cardiac and vascular malformations and embryos die around mid-gestation [55], whereas HIF-2*α* knockout mice die presumably of bradycardia due to an inadequate supply of catecholamines during embryonic development [56]. HIF-1*α* is present in mice under normoxic conditions, increases within distinct cell types in response to systemic hypoxia, and plays an important role in oxygen homeostasis [57]. HIF-2*α* plays an important role in cardiovascular development and angiogenesis [56, 58]. Murine embryonic stem cells lacking the HIF-2*α* gene revealed an association with the response to hypoglycemia rather than hypoxia, suggesting that HIF-2*α* may be more important in the survival response than oxygen level [59]. Hypoxia affects expression of the PER1 and CLOCK circadian genes, and HIF-1*α* interacts with PER1 under normoxic conditions [60]. The mouse Hif1a gene is expressed from two distinct promoter/first exon combinations, resulting in tissue-specific (mHIF-1alphaI.1) and ubiquitous (mHIF-1alphaI.2) forms. mHIF-1alphaI.1 is a novel HIF-1*α* mRNA isoform exclusively detected in elongated spermatids. The mHIF-1alphaI.1 protein is located in the mid-piece of the spermatozoal flagellum and expression is oxygen independent [61]. The mHIF-1alphaI.1 isoform is also upregulated in activated T cells under normoxic conditions, suggesting a physiological role for the mHIF-1alphaI.1 isoform in activated lymphocytes [62]. Interestingly, mice exposed to an elevated temperature strongly upregulate HIF-1*α* in the liver, kidney, and spleen, suggesting a novel mechanism to stabilize HIF-1*α* under normoxic conditions [63]. Furthermore, a recent study demonstrated that treating normal human cells with low-dose radiation induces a HIF-1-mediated adaptive and protective metabolic response and increased radiation resistance [64]. HIF-1*α* is induced and activated at physiological oxygen tensions in a mitogen activated protein kinase-dependent manner and determines the increased cell proliferation rate that is common under these conditions [65]. HIF-1*α* plays an important role in the adaptation of myocardium to mechanical stress via stress-activated channels [66]. As discussed previously, HIF-1*α* is required for mesenchymal cell survival, and HIF knockout mice have malformed cardiovascular systems and neural tube defects and die during mid-gestation [55, 67, 68]. Hypoxia potentiates interleukin (IL)-1*β* expression and attenuates selective targeting of IL-1*β* to autophagic degradation in activated macrophages, suggesting that a novel proinflammatory mechanism may participate in atherogenesis [69]. These observations support the notion that the HIF pathway plays important roles in physiological adaptation and is required for normal growth and development. Interestingly, mice with only one HIF-1*α* mutant allele develop normally but have impaired physiological responses to chronic hypoxia, such as reduced polycythemia, right ventricular hypertrophy, pulmonary hypertension, pulmonary vascular remodeling, and electrophysiological responses [70, 71]. The carotid body (CB) monitors arterial blood O_2_ levels and stimulates breathing in response to hypoxemia to ensure an adequate O_2_ supply. Both HIF-1*α* and HIF-2*α* are expressed in the CB [56, 72], and CB responses to hypoxia are impaired in* Hif1α+/−* mice [73], whereas they are exaggerated in* Hif2α+/−* mice [74]. Balanced HIF-1*α* and HIF-2*α* activity is critical for oxygen sensing by the CB and adrenal medulla and for their control of cardiorespiratory function [75]. Similarly, CBs isolated from HIF-1*α* heterozygous mice have a dramatic effect on neural activity and ventilatory adaptation after exposure to hypoxia, suggesting a role for HIF-1*α* at the systemic level [73]. Partial HIF-2*α* deficiency leads to increased levels of HIF-1*α* and NADPH oxidase 2, resulting in an oxidized intracellular redox state, exaggerated hypoxic sensitivity, and cardiorespiratory abnormalities, which are reversed by treatment with a HIF-1*α* inhibitor or a superoxide anion scavenger. In contrast, partial HIF-1*α* deficiency increases levels of HIF-2*α* and superoxide dismutase 2, leading to a reduced intracellular redox state, blunted oxygen sensing, and impaired CB and ventilatory responses to chronic hypoxia, which are corrected by a HIF-2*α* inhibitor. These observations demonstrate that the redox balance, which is determined by mutual antagonism between HIF-*α* isoforms, establishes the hypoxic sensing set-point by the CB and adrenal medulla and is required for maintenance of cardiorespiratory homeostasis [75]. HIF-2*α* plays an important role during the postvasculogenesis stages and is required to remodel the primary vascular network into a mature hierarchical pattern [58]. HIF-2*α* sense hypoxia during mid-gestational development and translate this signal into an altered gene expression pattern, leading to increased circulating catecholamine levels and proper cardiac function [56]. Despite of normoxic environment several tissues are inherently hypoxic suggesting importance of HIF pathway in normal development. Interestingly, tissue-specific targeting to delete HIF-1*α* in the cartilaginous growth plate of developing bone showed gross skeletal malformations and die perinatally, probably due to tracheal abnormalities suggesting the role of hypoxia in growth plate development [76]. In addition, hypoxic environment is essential for appropriate embryonic development and placentation. Oxygen tension determines whether cytotrophoblasts, specialized placental cells proliferate or invade, thereby regulating placental growth and cellular architecture [77]. Moreover, ARNT knockout mice placentas shows greatly reduced labyrinthine and spongiotrophoblast layers, and increased numbers of giant cells supporting that HIF-1*α* is essential for mammalian placentation [78]. Additionally, HIFs play important roles in modulating the developmental plasticity of stem cells by integrating physiological, transcriptional and epigenetic inputs in placenta [79]. Transforming growth factor (TGF) *β*-3, an inhibitor of extravillous trophoblast differentiation, partly mediates oxygen-regulated early events of trophoblast differentiation through HIF-1*α* pathway [80]. In addition, HIF-1alpha-deficient mouse embryonic fibroblasts showed impaired migratory capabilities and demonstrated that TGF-*β*-3 manifests hypoxia and HIF-1*α*-dependent regulation [81]. Furthermore, hypoxia signaling plays a central role in cartilage, bone, and hematopoiesis [82]. HIF-1*α* plays a bimodal role in cartilage homeostasis by enhancing anaerobic glycolysis and inhibiting apoptosis suggesting the potential role of this pathway in treatment of osteoarthritis [83, 84]. HIF-2*α*, was found to be essential for endochondral ossification of cultured chondrocytes and embryonic skeletal growth in mice and its function are independent of oxygen-dependent hydroxylation [85]. Elevated levels of HIF-1*α* promotes cartilage formation and maintenance whereas elevated levels of HIF-2*α* favors cartilage destruction and endochondral ossification [85, 86]. Taken together this suggests that both HIF-1*α* and HIF-2*α* plays an important role for normal growth and development of skeletal vasculature.

### 5.2. Regulation of Metabolic Pathways

Increased glycolysis during hypoxia is a crucial step to meet energy demands. Interestingly, HIF-1*α* stimulates glycolysis and actively represses mitochondrial function and oxygen consumption by inducing pyruvate dehydrogenase kinase-1 (PDK-1) activity [87]. That study also reported that PDK-1 phosphorylates and inhibits pyruvate dehydrogenase from using pyruvate to fuel the mitochondrial TCA cycle, which decreases mitochondrial oxygen consumption resulting in a relative increase in intracellular oxygen tension [87]. Another known mechanism to increase respiration efficiency in hypoxic cells is by regulating of cytochrome *c* oxidase (COX) activity. COX is located in the inner mitochondrial membrane and is a dimer composed of monomers with 13 subunits [88]. Subunits I, II, and III are encoded by the mitochondrial genome, constitute the catalytic core of the enzyme, and are highly conserved in eukaryotes. The crystal structure of bovine COX reveals that subunit IV (COX4) interacts with both COX1 and COX2 [88]. The first step of COX assembly in mammalian cells is the association of COX1 with COX4 [89]. COX4 binds ATP, within the complex, leading to allosteric inhibition of COX activity at high ATP/ADP ratios and demonstrating a regulatory role for COX4 [90]. Under hypoxic conditions, HIF-1*α* mutually regulates COX4 subunit expression by stimulating transcription of the genes encoding COX4-2 and LON, a mitochondrial protease required for COX4-1 degradation. The effects of manipulating COX-4 subunit expression on COX activity, ATP production, O_2_ consumption, and ROS generation indicate that the COX4 subunit switch is a homeostatic response that optimizes respiration efficiency at different O_2_ concentrations [11]. Simply, PDK-1 inhibits conversion of pyruvate to acetyl-CoA, thereby preventing pyruvate entry into the TCA cycle and the COX-4 subunits govern mitochondrial respiration efficiency in response to varied oxygen tensions [11, 87]. Autophagy may be the fourth adaptive metabolic response required to prevent increased ROS levels and cell death in hypoxic cells [91]. Mitochondria are replaced every 2–4 weeks in rat brain, heart, liver, and kidney [92]. The destruction of mitochondria is believed to occur via autophagy [93, 94]. Autophagy can be induced by environmental stress such as nutrient deprivation and provides a mechanism to dispose of damaged mitochondria [95, 96]. Autophagy is induced in the heart subject to hypoxic or ischemic conditions and has been proposed to play either a protective or pathogenic role in heart disease [96–98]. BNIP3 is an accepted HIF-1 target gene [99, 100] and is associated with autophagy [98]. BNIP3 may disrupt interactions between Beclin-1, a highly conserved protein required to initiate autophagy, and Bcl2 or Bcl-XL [101]. In contrast to HIF, c-MYC is a proto-oncogene that codes for a transcription factor, regulates the expression of 10–15% of all genes in the genome [102, 103], and promotes mitochondrial respiration by increasing biogenesis [103]. Under physiological conditions HIF-1*α* inhibits c-MYC activity by directly interacting and stimulating a proteasome-dependent pathway [104, 105]. c-Myc paradoxically collaborates with Hif-1 under stress conditions to induce PDK-1 and hexokinase 2 expression followed by aerobic glycolysis [105, 106] and angiogenesis [106].

### 5.3. Role of the HIF Pathway in Cell Proliferation and Cell Death

The role of HIF pathway in cell death is controversial; HIF-1 can induce apoptosis [107], prevent cell death, or even stimulate cell proliferation [108]. The oxygen concentration determines whether cell will go apoptosis or not; oxygen level in the range 0–0.5% could induce apoptosis whereas cells with oxygen levels in the range of 1–3% do not undergo apoptosis [109]. ATP is another key determinant of apoptosis; as long as cells have an enough supply of glycolytic ATP during oxygen deprivation, apoptosis can be executed [110]. Moreover, lack of oxygen inhibits the electron transport chain at the inner membrane of the mitochondria thereby causes a reduction in the membrane potential. This reduction of mitochondrial derived ATP causes activation of Bax or Bak, leading to cytochrome C release into the cytosol [111]. Cytochrome c is released and caspase-9 is activated in oxygen-deprived cells undergoing apoptosis [110, 111]. Furthermore, p53 protein, an important regulator of apoptosis can induce the Bax and Bak proteins thereby initiating the cascade leading to apoptosis through cytochrome C [112]. Interestingly, fibroblasts from mice lacking both Bax and Bak genes are resistant to oxygen deprivation induced apoptosis [110]. Similarly, cell over-expressing Bcl-2 or BcL-XL, the anti-apoptotic proteins prevents oxygen deprivation induced apoptosis by inhibiting the release of cytochrome c from the mitochondria [110, 113, 114]. In addition to energy deprivation, ROS generation contributes to hypoxia induced apoptosis. In this case, the initiator caspase 9 is cleaved directly to the active form by caspases 3 and 12, without the involvement of cytochrome C in response to hypoxia [115]. Activation of c-Jun NH2-terminal kinase (JNK) is another mechanism by which hypoxia can induce apoptosis in melanoma cells [116].

HIF pathway is also involved in cell proliferation and promoting metastases [117, 118], VEGF is particularly noteworthy target gene of HIF pathway involved in cell proliferation and upregulated in most cancers [119, 120]. The HIFs can alter cell-cycle progression through putative transcriptional targets such as Cyclin D1 [121] and indirect modulation of p21 and p27 [122]. Furthermore, hypoxic induction of HIF-1*α* suppresses cell proliferation and acute HIF-1*α* stabilization at moderate hypoxia (1% O_2_) results in cell cycle arrest by inhibiting c-Myc transcriptional activity [122]. In contrast, HIF-2*α* induction promotes cell cycle progression by enhancing c-Myc function [105].

### 5.4. Role of the HIF Pathway in pH Regulation and Exercise

The correlation between hypoxia and intracellular pH (pHi) was extensively researched in the 1980–90s. Rapid build-up of intracellular lactate and H^+^ as well as an extensive decrease in phosphocreatine concentration is the first sign of hypoxia. ATP is more resistant to hypoxia provided that glucose is present. Furthermore, metabolic damage is considerably greater if glucose is absent during the insult, suggesting that either anaerobic ATP production or low pH may exert some protective effect against acute cell damage [123]. The regulation of Na^+^/H^+^ exchange (NHE) and pHi is vital for maintaining cell viability. pHi modulates a number of important cell functions, including signal transduction pathways involved in the regulation of cell size and proliferation [124–126]. Alterations in pHi are also associated with hypoxic pulmonary vasoconstriction [127, 128]. Pulmonary arterial smooth muscle cells from chronic hypoxic mice have an elevated basal pHi accompanied by an increase in NHE activity, secondary to increased NHE1 isoform expression [129]. HIF-1*α* plays an important role governing increased NHE, NHE1 expression, and alkalinization of pulmonary arterial smooth muscle cells in response to hypoxia [130]. Interestingly, NHE inhibitors attenuate hypoxia-induced apoptosis in cardiac myocytes [131]. Tumor cells have a lower extracellular pH (pHe) and a higher pHi than those of normal cells. Low pHe promotes invasiveness, whereas high pHi provides a competitive advantage to growth of tumor cells compared to normal cells for [132, 133]. Low pHe results from lactic acid accumulation solely produced by glycolysis. Hypoxia induces coordinated upregulation of glycolysis, a potential step that may promote tumor cell growth and activate the capacity of tumor-associated carbonic anhydrase (CA) IX to acidify pHe thereby leading to tumor progression [134]. HIF-1*α* controls CA IX and CA XII expressed by tumor cells. Hypoxia-inducible CAIX and CAXII proteins promote cell survival and growth by maintaining pHi. Moreover, CAIX and CAXII constitute a robust pHi-regulating system that confers a tumor growth and survival advantage to cells exposed to a hypoxic and acidic microenvironment [135].

HIF is a candidate to facilitate training adaptation in skeletal muscle. Muscle training induces negative regulators of HIF (PHD, FIH, and sirtuin-6) but lowers PDK-1 expression in elite athletes, thereby contributing to skeletal muscle adaptation to exercise [136]. Endurance exercise improves muscle oxidative capacity [137], whereas resistance exercise training leads to increased muscle size and strength [138]. One study investigated the effects of 8 weeks of resistance exercise training performed under hypoxic (HRT) or normoxic conditions (NRT) on skeletal muscle. As results, significant increases in muscle endurance, plasma VEGF concentration, and capillary-to-fiber ratio were observed following training in the HRT group compared those in the NRT group, suggesting that HRT may also lead to increased muscular endurance and promote angiogenesis in skeletal muscle [139]. Chuvash polycythemia (CP) is an autosomal recessive disorder in which regulatory degradation of HIF is impaired, resulting in elevated levels of HIF under normal oxygen tensions [140, 141]. Patients with CP show early and marked phosphocreatine depletion, higher blood lactate accumulation, acidosis in skeletal muscles, and reduced exercise capacity [142]. Interestingly, gene therapy with intramuscular administration of Ad2/HIF-1*α*/VP16 was not effective for patients with intermittent claudication [143]. Pyruvate dehydrogenase (PDH) plays an important role controlling the flux of pyruvate to acetyl-CoA. PDH is inactivated during acute hypoxia thereby promoting conversion of pyruvate to lactate, suggesting an influence of PDH activity on the fate of pyruvate [87]. The transition from acute to chronic hypoxia desensitizes the HIF-1*α* pathway, leading to a re-establishment of PDH activity and reduced lactate production by exercising muscles [144]. Exercise with intermittent hypoxic training for 3 weeks causes a significant decrease in skeletal muscle HIF-1*α* mRNA, suggesting that transcriptional and posttranscriptional regulation of the HIF-1 differ in muscle and other cells [145]. HIF-1*α* and HIF-2*α* induce angiogenesis and improve muscle energy recovery [146]. In contrast, the HIF-3*α* subunit plays a negative role in adaptation to hypoxia because inhibiting HIF-3*α* expression leads to increased physical endurance [147].

## 6. Conclusion

In present review we have discussed the physiological adaptations and importance of the HIF pathway. Several studies have demonstrated that manipulating the HIF pathway can help treat diverse disorders. HIF(s) are also upregulated under inflammatory conditions, suggesting their role in maintaining homeostatic conditions and protecting against cellular inflammation. The role of the HIF pathway varies under diverse conditions. For example, HIF inhibitors have been developed to treat cancer and ischemia, whereas HIF activators could be utilized for stroke and spinal cord injuries. Significant developments have been made towards understanding the roles of the HIFs under both physiological and pathophysiological conditions. The roles of HIF(s) are becoming clearer during physiological adaptation. The interaction of HIF-1*α* with HIF-2*α* and HIF-3*α* during physiological adaptation will provide a great deal of understanding of HIF(s). Moreover how these transcription factors interact with other known proteins and pathways will help in designing the future therapeutics with minimal side effect.
